# A mobile device app to reduce prehospital medication errors and time to drug preparation and delivery by emergency medical services during simulated pediatric cardiopulmonary resuscitation: study protocol of a multicenter, prospective, randomized controlled trial

**DOI:** 10.1186/s13063-019-3726-4

**Published:** 2019-11-20

**Authors:** Johan N. Siebert, Laurie Bloudeau, Frédéric Ehrler, Christophe Combescure, Kevin Haddad, Florence Hugon, Laurent Suppan, Frédérique Rodieux, Christian Lovis, Alain Gervaix, Sergio Manzano

**Affiliations:** 1 Children’s Hospital, Department of Pediatric Emergency Medicine, Geneva University Hospitals, 47 Avenue de la Roseraie, 1211 Geneva 14, Switzerland; 2 A.C.E. Geneva Ambulances SA, 2 Route de Jussy, 1225 Geneva, Switzerland; 3 Division of Medical Information Sciences, Department of Radiology and Medical Informatics, Geneva University Hospitals, 4 Rue Gabrielle Perret-Gentil, 1211 Geneva 14, Switzerland; 4 Division of Clinical Epidemiology, Department of Health and Community Medicine, University of Geneva and Geneva University Hospitals, 4 Rue Gabrielle Perret-Gentil, 1211 Geneva 14, Switzerland; 5 Department of Emergency Medicine, Geneva University Hospitals, 4 Rue Gabrielle Perret-Gentil, 1211 Geneva 14, Switzerland; 6 Service of Clinical Pharmacology and Toxicology, Geneva University Hospitals, 4 Rue Gabrielle Perret-Gentil, 1211 Geneva 14, Switzerland; 7 Geneva University Faculty of Medicine, 1 Rue Michel Servet, 1205 Geneva, Switzerland

**Keywords:** Resuscitation, Medication errors, Pharmaceutical preparations, Biomedical technology, Mobile applications, Emergency medical services, Pediatrics

## Abstract

**Background:**

Emergency drug preparation and administration in children is both complex and time-consuming and places this population at a higher risk than adults for medication errors. Moreover, survival and a favorable neurological outcome from cardiopulmonary resuscitation are inversely correlated to drug preparation time. We developed a mobile device application (the pediatric Accurate Medication IN Emergency Situations (PedAMINES) app) as a step-by-step guide for the preparation to delivery of drugs requiring intravenous injection. In a previous multicenter randomized trial, we reported the ability of this app to significantly reduce in-hospital continuous infusion medication error rates and drug preparation time compared to conventional preparation methods during simulation-based pediatric resuscitations. This trial aims to evaluate the effectiveness of this app during pediatric out-of-hospital cardiopulmonary resuscitation.

**Methods/design:**

We will conduct a multicenter, prospective, randomized controlled trial to compare the PedAMINES app with conventional calculation methods for the preparation of direct intravenously administered emergency medications during standardized, simulation-based, pediatric out-of-hospital cardiac arrest scenarios using a high-fidelity manikin. One hundred and twenty paramedics will be randomized (1:1) in several emergency medical services located in different regions of Switzerland. Each paramedic will be asked to prepare, sequentially, four intravenously administered emergency medications using either the app or conventional methods. The primary endpoint is the medication error rates. Enrollment will start in mid-2019 and data analysis in late 2019. We anticipate that the intervention will be completed in early 2020 and study results will be submitted in late 2020 for publication (expected in early 2021).

**Discussion:**

This clinical trial will assess the impact of an evidence-based mobile device app to reduce the rate of medication errors, time to drug preparation and time to drug delivery during prehospital pediatric resuscitation. As research in this area is scarce, the results generated from this study will be of great importance and may be sufficient to change and improve prehospital pediatric emergency care practice.

**Trial registration:**

ClinicalTrials.gov, ID: NCT03921346. Registered on 18 April 2019.

## Background

Children are a vulnerable population with specific medical needs compared to adults. The fast, accurate and safe preparation and administration of intravenously administered (IV) drugs is both complex and time-consuming in pediatric critical situations, such as cardiopulmonary resuscitation (CPR) [1–4]. Most drugs given intravenously to children are provided in vials originally prepared for the adult population. This leads to the need for a specific, individual, weight-based drug-dose calculation and preparation for each child that varies widely across age groups [3, 5–9]. This error-prone process and the lower dosing error tolerance of children [10] places them at a high risk for life-threatening medication errors [3, 5, 6, 11]. Despite well-equipped and staffed environments with numerous available safeguards, direct IV medication errors have been reported in up to 41% of cases during simulated in-hospital pediatric resuscitations, 65% of which were incorrect medication dosage, thus making it the most common error [12]. The rate of errors is also important in the high-risk prehospital setting, which is reported as occurring in more than 30% of all pediatric drugs administered, with an error rate for epinephrine dosage alone of more than 60% [13]. In this particular context, initial care has to be delivered quickly by emergency medical services (EMS) in challenging field environments where resources and providers are limited [14]. A single paramedic is often in charge of determining the patient’s weight, choosing the most suitable drug, calculating the drug dose and appropriate volume to inject, and administering it to the patient. However, as paramedics have little exposure to pediatric education during their initial training [15] and, thereafter, to critically ill children during their work hours [16], they have limited opportunities to administer resuscitation drugs at pediatric doses and to improve their skill level.

In resuscitation, time is a critical success criterion. During the first 15 min of pediatric CPR, survival and favorable neurological outcome decrease linearly by 2.1 and 1.2% per min, respectively [17], and rely in part on drug preparation time in both in-hospital [18] or out-of-hospital settings [19]. Among non-shockable pediatric out-of-hospital cardiac arrests, each minute delay to epinephrine delivery is associated with a 9% decrease in survival odds [19, 20]. Regrettably, most patients in the prehospital setting receive epinephrine more than 10 min after EMS arrival [19, 20]. Therefore, the chain of survival critically relies on early out-of-hospital CPR by EMS [21] and on-site administration of emergency drugs without delay [19, 20, 22] before a rapid transfer to pediatric emergency departments (PED) and advanced care. Despite efforts to solve this problem, out-of-hospital preparation and delivery of pediatric emergency drugs remain a worldwide health challenge. The evaluation of new methods to reduce pediatric medication errors is of paramount importance, but research in this area is scarce.

### Previous work justifying this trial

In a previous multicenter, randomized crossover trial, we showed that medication errors, time to drug preparation, and time to drug delivery for continuous infusions during simulation-based, pediatric, in-hospital post-cardiac-arrest scenarios were significantly reduced by using a mobile device app (the pediatric accurate medication in emergency situations (pediatric Accurate Medication IN Emergency Situations (PedAMINES)) designed to help pediatric drug preparation [23].

### Objectives

The primary aim of this multicenter study protocol is to compare the impact of the app with conventional calculation methods for the preparation of direct IV drugs during standardized, simulation-based, pediatric out-of-hospital cardiac arrest scenarios. We hypothesized that the use of the app might extend and scale-up our previous multicenter in-hospital observations by similarly reducing the occurrence of medication errors and time to drug preparation and delivery when used in out-of-hospital settings by paramedics, independent of EMS skills.

## Methods/design

### Trial design

We will conduct a prospective, multicenter, randomized controlled trial with two parallel groups in several EMS located in different regions of Switzerland, a pluralistic country with four official languages without uniformly standardized or benchmarked EMS clinical guidelines, protocols or operating procedures. Participants allocated to the conventional preparation method group will be allowed to use a calculator, but not any other drug preparation support enabling weight-based drug-dose calculation, such as an online calculator or a mobile device app. The final correct volume of drugs to be drawn will not be released to the paramedics. To calculate the volume of drug to inject, the desired drug to be delivered in milligrams is first selected from a calculation of the original weight-based prescription in mg/kg. The next step is to convert the milligrams into milliliters of drug to be drawn. For the purpose of the study, we will not select drugs that can be directly drawn from the vial without calculation. Participants allocated to the app group will not be allowed to use any other drug preparation support.

**Fig. 1:**
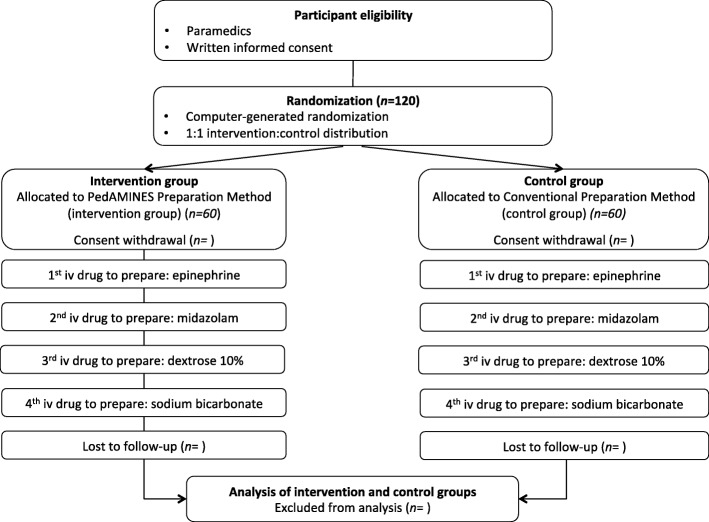
Trial flow chart

**Fig. 2:**
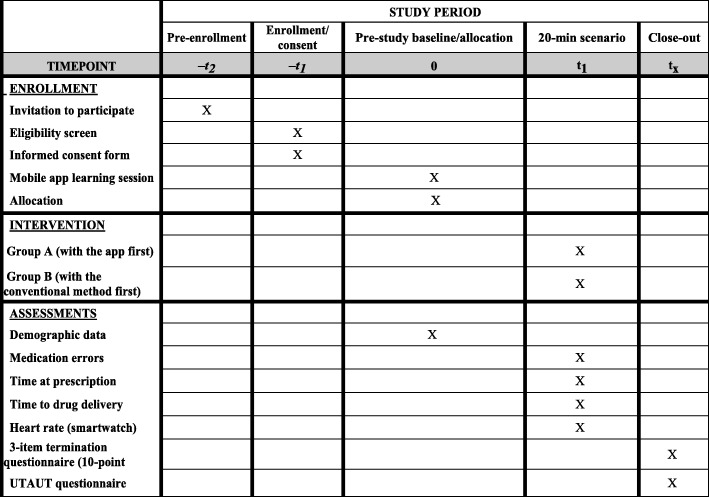
Standard Protocol Items: Recommendations for Interventional Trials checklist (SPIRIT) Figure

Figure 1 shows the trial flow chart and Fig. 2 the trial schedule. The study will be carried out in accordance with the Consolidated Standards of Reporting Trials of Electronic and Mobile Health Applications and Online TeleHealth (CONSORT-EHEALTH) [24] guidelines and the Reporting Guidelines for Health Care Simulation Research [25]. The present study protocol adheres to the Standard Protocol Items: Recommendations for Interventional Trials (SPIRIT) 2013 Checklist (Additional file 1) [26].

The trial protocol received a declaration of no objection by the Geneva Cantonal Ethics Committee on 29 March 2018. The trial will be conducted according to the principles of the Declaration of Helsinki [27] and Good Clinical Practice guidelines [28]. Results will be reported according to recommendations in the CONSORT-EHEALTH Statement [24] and the Reporting Guidelines for Health Care Simulation Research [25]. It is our intention to present these at scientific congresses and to publish the results in an international peer-reviewed journal, irrespective of the magnitude or direction of effect.

### Participants

Registered paramedics working in Swiss EMS are eligible for inclusion in the study. Inclusion criteria are having followed a standardized 5-min introductory course on the use of the mobile device app and being willing to grant written informed consent. They will be excluded if they had previously used a numerical device aimed at helping with drug preparation. All participants will be assumed to have an equivalent competence with direct IV drug preparation and dose calculation as this is part of their regular practice and training background.

### The PedAMINES app

**Fig. 3:**
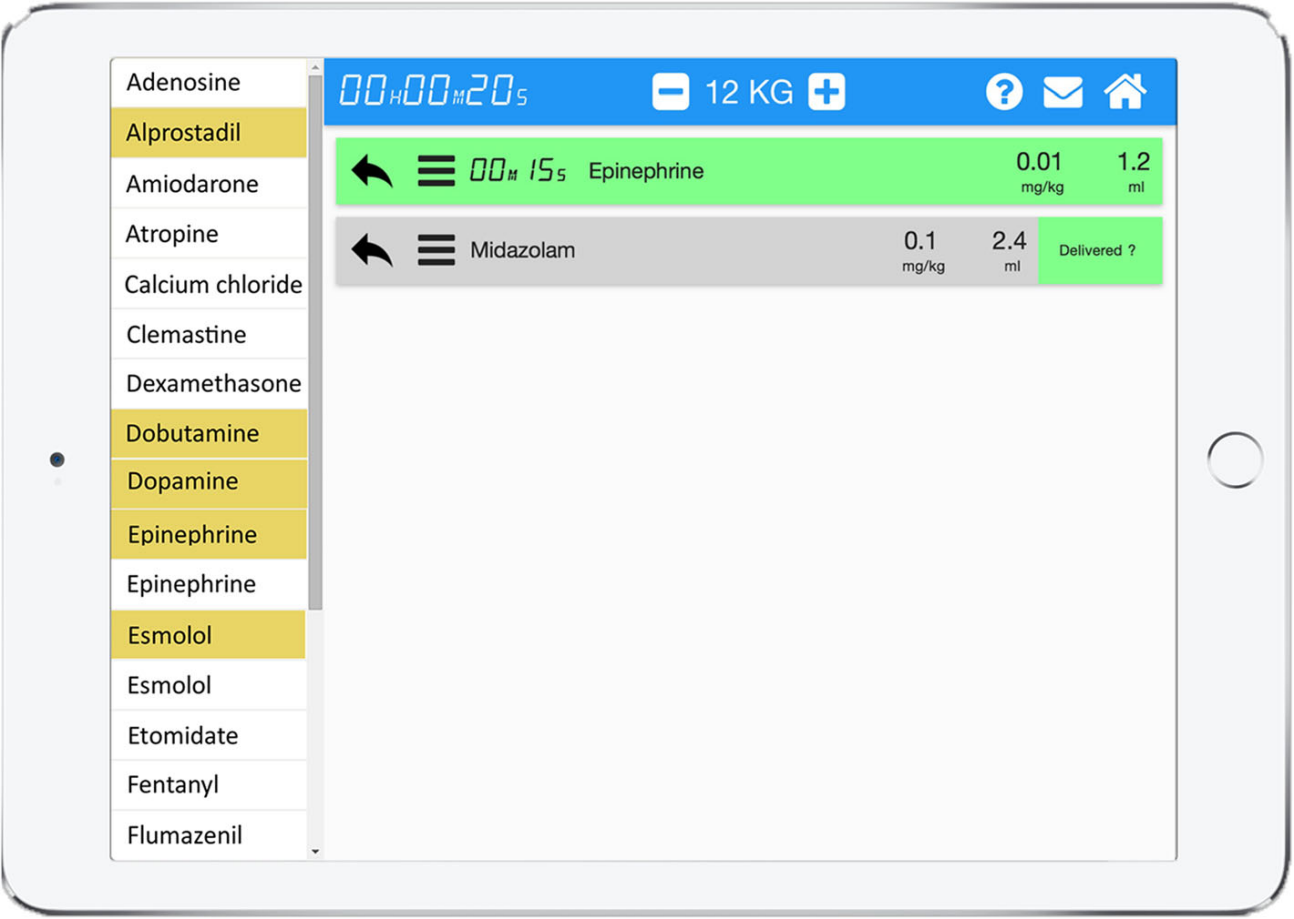
Pediatric Accurate Medication IN Emergency Situations (PedAMINES) screenshot. List of bolus intravenously administered (IV) drugs (white boxes) and drugs for continuous infusion (yellow boxes) are selectable in the left margin of the application. The right window shows drugs selected by the paramedic for a child weighing 12 kg. In this screenshot example, epinephrine is being delivered at 0.01 mg/kg (0.1 mL/kg of 0.1 mg/mL concentration). Midazolam 0.1 mg/kg (of 5 mg/mL concentration and 10 mL sodium chloride 0.9%) is selected and ready to be injected, waiting for the nurse’s approval (“delivered?”). The printer logo in the upper right corner indicates that all actions performed by the nurses are sequentially saved in historic files that can be retrieved and printed at any time

The app was developed at Geneva University Hospitals (Geneva, Switzerland) following a user-centered and evidence-based approach with emergency department caregivers, software developers and ergonomists. On the basis of pediatric resuscitation observations and focus groups, the team worked closely together to identify the key functionalities and processes to be implemented [29]. The app lists all the available resuscitation drugs for either direct IV injection or continuous infusion with doses automatically adapted to the weight or age of the patient based on information entered when starting the app. With one touch, any of the listed drugs can be selected and shown with a detailed preparation according to a standardized and simplified pathway. In the case of a direct IV injection, this pathway is composed of two steps: (1) drug selection and (2) conversion of the prescribed dose in mg/kg into a volume in milliliters. If necessary, an additional step is provided for the dilution of the initial drug concentration with compatible fluids (sodium chloride 0.9%, etc.). For each drug, the exact amount to prepare is clearly displayed and thus avoids the need for calculations (see screenshot, Fig. 3). This is based on the app’s ability to automatically calculate the optimal weight-based final volume to inject and describe the preparation sequence required to achieve it, independent of the user’s competency in this domain. When using the app, the user can interact with it at any time. Multiple drugs can be prepared and run in parallel, including continuous infusions. All actions by the user are sequentially saved locally on the device in historic files to preserve information that can be retrieved at any time for debriefing or medicolegal purposes. Historic files can also be erased or safely exported and saved in electronic health records.

### Intervention and resuscitation scenario

On the day of participation after random allocation, each participating paramedic will: (1) complete a survey collecting data regarding their demographics, care training, and simulation and computer experience; (2) receive a standardized 5-min training session on how to use PedAMINES; as well as (3) a presentation of the simulation manikin characteristics. The paramedics will then be asked to perform a 20-min highly realistic pediatric CPR scenario on a high-fidelity WiFi manikin (Laerdal SimBaby, Laerdal Medical, Stavanger, Norway). The procedure will be standardized across all sites to follow the same chronological progression and range of difficulty in order to ensure that each participant is exposed to exactly the same case, with similar challenges in decision-making and treatment preparation provided on the same manikin. The uniform delivery of the scenario throughout the entire study will minimize confounders. Study team members will only adapt to the progression speed of participants through the scenario by maintaining a stressful resuscitation atmosphere. The scenario will be conducted in an out-of-hospital simulated child’s bedroom environment to increase realism. High levels of realism are known to immerse participants in the simulated experience and prevent confounding variables that might potentially affect the way that individuals perform [30]. The room will be exclusively devoted to the simulation to prevent unexpected interruptions or external stimuli. Portable monitoring alarms will be activated to increase realism and stress. The scenario will be filmed with three action video cameras (GoPro, Hero 5 and 7 Black edition; San Matteo, CA, USA) worn by the participating paramedic and placed within the room.

The untimed portion of the simulation will involve a resuscitation team comprised of the same two study team members throughout the whole study period. One member (LB) will play the role of a second paramedic leading the CPR and assisting the study participant by performing chest compressions and bag-valve mask ventilation, but not drug-dose calculation or preparation, and the other (JS) will have the role of an advanced life-support physician as part of the responding crew, but supposedly dispatched to the scene in a second phase to prescribe the emergency drugs. In several countries, physicians are an integral part of prehospital EMS teams and are often dispatched to the most severe cases, including cardiac arrest [31]. Drug prescription will comply with standard international pediatric life-saving doses. A certified technician (SM) will operate the simulator and a fourth study investigator (KH) will play the role of the patient’s father, supposedly devoid of resuscitation knowledge and competencies. Participants will be informed before the scenario starts that these four people are study team members. The second paramedic will guide each participant through a series of predefined key steps, blinded to the participant, following a standardized resuscitation scenario (see below). The physician will order sequentially the medications using International Non-proprietary Names and allow progression through the scenario only once predefined milestones have been reached, irrespective of error occurrence or the time taken to achieve them. Study-specific training and standardization of the second paramedic and physician is ensured through their involvement in the previous in-hospital studies [23, 32] and by following the predefined scenario.

The untimed portion of the simulation will start by turning on the three video cameras and a fitness watch on the participant’s wrist, with both paramedics waiting outside the room. Both will be invited to enter the child’s bedroom by the patient’s father. When entering the room, a clinical statement to recognize the life-threatening condition of the patient, including his exact weight and age, will be given by the father as follows: “Here is Junior, a 12-kg, 18-month-old boy who suddenly collapsed 15 minutes ago. Oral pills belonging to his grandmother were found in his mouth and on the floor of his room. He is unconscious, pale and not breathing.” Looking at the empty medicine boxes, the second paramedic says “that the pills are an oral tricyclic antidepressant, as well as antidiabetic medication.” At this moment, the second paramedic says: “OK, I’ll take the lead of the resuscitation,” and asks the participant to take a central pulse. Due to the invariable absence of a pulse, the participant is asked to assist the leader in doing a 2-min full-course massage and ventilation (30:1 ratio) maneuver, with the massage carried out by the participant to increase their stress level. During this time, the leader places a supraglottic airway device in the manikin’s throat and the defibrillator patches on the trunk. The physician then enters the room and an asystole rhythm is recognized and verbalized. Both the physician and leader rotate the person performing the massage-ventilation maneuvers (new 15:2 ratio), ask the participant to place a vascular access on the manikin’s right hand (not intra-osseous to preserve the manikin integrity; first IV attempt successful) and then to prepare the drugs.

On the basis of the American Heart Association pediatric cardiac arrest algorithm for asystole [33], a bolus of 0.01 mg/kg epinephrine (0.1 mL/kg of 0.1 mg/mL concentration) is ordered by the physician and the timed scenario begins. The participating paramedic must prepare and administer the drug with the help of the app (intervention group) or following the conventional calculation method. The return of spontaneous circulation ensues. At this time, an upper-arm blood pressure monitor, a digital pulse oximeter on the right index finger, and a capnograph on a bag-valve mask are placed on the manikin who suddenly begins to have generalized tonic-clonic seizures. The physician says “the patient has now a return of spontaneous circulation with a pulse, but with seizures” and tells the participant “this patient needs a direct IV bolus of 0.1 mg/kg midazolam (of 5 mg/mL concentration and 10 mL sodium chloride 0.9%) right now,” while the leader is invited to stop the massage-ventilation maneuvers. The seizures stop 15 s after administration of the drug. At this time, the physician asks the leader to perform a fingerstick blood sample. The glucometer reports a blood sugar of 0.8 mmol/L. The physician says “the patient has a severe hypoglycemia” and prompts the participant to prepare and inject a direct IV bolus of 4 mL/kg dextrose 10%. Return of a state of consciousness ensues with normal vital signs, but a wide QRS complex on electrocardiogram monitoring. The physician says “this child needs a direct IV bolus of 1 mmol/kg sodium bicarbonate (of 4.2% = 0.5 mmol/L concentration).” As soon as this last medication is administered, the physician asks for transport to advanced hospital care and the scenario ends. The GoPro cameras and the watch are turned off 1 min later.

During the timed scenario, the resuscitation team will maintain a stressful resuscitation atmosphere by frequently reporting vital signs aloud and asking the participant to promptly provide the drugs, the monitoring alarms will be turned on, and the father will repeatedly verbalize his dismay. The measured deviation between the amount of drug delivered and the actual prescribed dose will be measured by the amount of drug in the syringe and video-recorded. All usual EMS resuscitation equipment will be at the disposal of the paramedic. In both allocation groups, the decision to use or not use any equipment will remain personal as in real life. Neither pilot testing nor repetitions will be permitted. There will be no interventions or educational adjuncts prior to or after the study period. To ensure that participants hear and understand the prescription orders correctly and to avoid comprehension bias, they will have to confirm the orders verbally and written transcriptions will be checked and video-recorded. Immediately after the scenario, participants will be asked to recall and describe precisely how they had prepared the drugs and to complete a questionnaire about the scenario.

### Outcomes

The primary outcome will be the proportion of medication dosage containing errors that occur during the sequence from drug preparation to drug injection. We define an emergency medication-dose administration error as a deviation from the correct weight dose of more than 10% [7]. These errors will be measured both as the percentage deviation from the amount of delivered drug compared with the correct weight dose as prescribed by the physician and the absolute deviations from that dose. Miscalculation of the final drug amount and the inability to calculate drug dosage without calculation and guidance help from the second paramedic will also be considered as medication errors. The accumulation of some or all of these errors will be defined as a cumulative error.

The secondary outcome will be the elapsed time in seconds between the oral prescription by the physician and time to drug preparation completion by the participant, the elapsed time in seconds between the oral prescription by the physician and the time to drug delivery by the participant (both times being relevant temporal values described in the pediatric resuscitation literature [17, 19, 34], and analysis of the type of medication error (i.e., error in transcription of the physician’s order into the medication dose, wrong choice of drug, wrong vial’s initial concentration, wrong air purge out of the syringe before injection, stage of error detection (before or after injection), and aseptic errors) (Additional file 3). These measures of medication errors have been selected as they were considered to be the most commonly reported in the pediatric medication-error literature and a meta-analysis [35–37]. In addition, a three-item questionnaire using a 10-point Likert scale will be administered to participants. The questionnaire measures: (1) the stress perceived before the scenario starts (“on a scale from 1 to 10, how stressed are you now?”); (2) the overall stress perceived at the end of the scenario (“on a scale of 1 to 10, how stressed (maximum reached) were you during the drug preparation period?”); and (3) the satisfaction about the preparation method used during the resuscitation scenario (“on a scale of 1 to 10, how satisfied were you with your preparation experience?”).

The participants’ stress level will be also assessed by measuring continuously their heart rate (HR) using a smartwatch on their wrist during the resuscitation scenario. The baseline HR will be recorded 1 min before the scenario starts. Mean delta HR values (difference between HR peak values and baseline HR) will be obtained during some small segments of scenario and correlated to the scenario phases and the preparation methods used. The segments of interest are: (1) when prompted to start the resuscitation just before massage and ventilation; (2) when prompted to prepare each drug; (3) the first 30 s when each drug is being prepared; (4) the last 30 s when each drug is being prepared; (5) upon the announcement of successful resuscitation achievement; and (6) 1 min later.

Acceptability and usability testing of the app will be assessed using a 52-item questionnaire based on the unified theory of acceptance and use of technology (UTAUT) model [38]. The UTAUT is a standardized instrument for measuring the likelihood of success of new technology introductions and helps to understand the drivers of its acceptance.

### Methods of measurement and data collection

Research using simulation as a valid and reliable investigative methodology to study factors affecting human and systems performance in health care has been reviewed [30]. In this study, all actions (i.e., outcomes) performed by the paramedics during the scenario will be automatically recorded and stored by the responsive simulator detectors (Laerdal SimBaby, Laerdal Medical, Stavanger, Norway) and the three GoPro video cameras. The set-up of the three cameras will be standardized to record at a resolution of 1080p at 90 frames per second, a wide field of view, and a 16:9 aspect ratio. Similarly, the position of the cameras will be standardized. The first camera will be mounted on a head strap placed on the paramedic’s head with a 45° downward inclination to allow to capture footage of the front scene. The second camera will be placed on a tripod in front of the paramedic and the manikin, slightly above head height, with a 90° downward inclination to film the place where the drugs will be prepared. The third camera will be placed on a tripod 1 m away from the paramedic on their left (if right-handed) or right (if left-handed) at the navel level to film the scene from the side. The recorded videos will be safely stored in triplicate on secured hard-disk drives, kept in a locked room, and centralized at the Children’s Hospital in Geneva. As all scenarios will be fully video-recorded, medication errors and any other errors will be recorded and later analyzed.

All actions performed with the app will be automatically saved locally in log files for further analysis. The validity and reliability of the app has been assessed in prior studies [23, 32]. The stress level (HR) of each participant will be recorded during the entire resuscitation scenario with the HR monitor on a Polar A360 watch (Polar Electro Oy, Kempele, Finland). Data will be stored on the wristwatch itself with further analysis performed offline. The investigators will double-check on-site that the questionnaires are fully and accurately completed. Data collection will be carried out using the REDCap database (REDCap, Vanderbilt University, Nashville, TN, USA). This study offers the major advantage to observe a unique 60-min period per paramedic. Therefore, neither follow-up nor retention plans will be necessary. The intervention protocol is highly standardized and paramedic deviation from the protocol in terms of drug preparation is a parameter that is of interest in our study (i.e., in terms of medication errors or delays in drug preparation).

### Power and sample size calculation

The expected proportion of errors made by EMS without PedAMINES is 60% [6]. The sample size was calculated to provide the trial with 90% power at a two-sided alpha level of 5% in detecting an absolute difference of at least 30% in proportions of medication errors between intervention groups. The required sample size is 56 paramedics per study arm. To prevent a potential loss of power due to misspecification of assumptions, 60 paramedics will be recruited per randomized group (total sample size: 120 paramedics). To achieve adequate participant enrollment to reach the target sample size, shift-working paramedics will be randomly recruited weeks before the start of the study by a blinded non-investigator. They will be informed of the upcoming simulation study but not of its purpose and outcomes.

### Group allocation

Paramedics will be randomized using a stratified, single, constant 1:1 allocation ratio determined with web-based software [39]. One randomization list per EMS center will be produced (randomization stratified on center) and random block sizes will be used to generate the randomization lists. On the day of participation, each participant and an investigator will sign the informed consent (Additional file 2), and selection criteria will be checked prior to participation in the study.

### Blinding

Blinding to the direct IV drugs and doses intended for use will be maintained during recruitment to minimize preparation bias. Allocation concealment will be ensured with the allocation software and will not be released until the paramedics start the scenario. Study team members will be revealed to the participants just before the scenario starts. Although the intervention could not be masked, all investigators will remain unaware of the outcomes until all data are unlocked for analysis at the end of the trial. All scenarios will be video-recorded for later analysis. A post-scenario video review will be done without blinding by two reviewers, but undertaken independently with each blinded to the other’s reviews. In the case of disagreement, a third independent evaluator will help reach a consensus. The data analyst (CC) will be blinded to treatment allocation.

### Confidentiality

Information about study subjects will be kept confidential. All data will be entered into the REDCap data management system where all data on study subjects are assigned an individual identifying code that does not contain identifying information.

### Statistical analyses

For the primary outcome, the proportion of medication errors will be reported for each method and by each type of medication (epinephrine, midazolam, dextrose 10%, sodium bicarbonate), with the exact Clopper-Pearson 95% confidence interval (CI). All differences in medication error rates between preparation methods will be reported with exact 95% CIs. The association between the preparation methods (app versus conventional method) and the risk of error will be assessed using a logistic regression model with mixed effects to account for the repetition of measures among paramedics. A random intercept will be introduced in the model. The model will be adjusted for the type of medication as the risk of error can vary across medications, and by center as the randomization procedure is stratified on centers.

For the secondary outcomes, time to drug preparation and time to drug delivery will be reported for each method and by each type of medication (epinephrine, midazolam, dextrose 10%, sodium bicarbonate), with the 95% CI. The mean difference between preparation methods will also be reported with 95% CI. The association between the preparation methods and time to drug preparation and time to drug delivery will be assessed by using a linear regression model with mixed effects to account for the repetition of measures among paramedics. A random intercept will be introduced in the model. The model will be adjusted for the type of medication since time to drug preparation and time to drug delivery can vary across medications. In addition, the model will be adjusted by center as the randomization procedure is stratified on centers. The type of medication errors will be analyzed in a similar manner to the primary outcome. For each of the four drugs prepared, errors will be measured by the deviation from the amount of drug taken, the amount of saline solution taken to dilute the drug if needed, the final concentration, and the correct weight-based dose. Absolute deviation will be analyzed for each type of medication. The mean (or median) difference in deviation obtained with the app and the conventional method will be reported with 95% CI (or interquartile range). A linear regression model adjusted for centers or a Van Elteren test stratified by center will be used to compare preparation methods.

For primary and secondary outcomes, regression analyses will be conducted if applicable to test a difference in error rates between an urban EMS (defined as a primary EMS in an area populated with 50,000 or more people in the immediate proximity of a tertiary care PED) [40] and a rural EMS (EMS agency not included within an urban area) with the app and conventional methods. In regression models with mixed effects, an interaction between interventions and urban/rural EMS will be tested to investigate a potential modification of the efficacy of the app in an urban area compared with a rural area. Results will be also correlated to the EMS exposure (i.e., total number of emergency calls per year per EMS divided by the number of paramedics working in that EMS). Analyses of primary and secondary outcomes will be also conducted with both preparation methods according to paramedics’ experience, expressed as years since certification.

A first reviewer will review all videos. To assess the reproducibility of the video review procedure, a second reviewer will independently duplicate the review in a random sample of 10% of all videos. Interrater reliability scores on video reviewing will be calculated using Cohen’s kappa coefficient for the medication errors. We define poor reliability as a kappa coefficient of < 0.4, fair reliability as 0.4–0.6, good reliability as > 0.6–0.8, and excellent as > 0.8. Through previous training in video reviewing of similar outcomes during simulation-based pediatric resuscitation [23], we will ensure that both reviewers have a high level of interrater reliability with kappa values for primary outcome measure of at least 0.6, prior to beginning the trial. Any disagreements in video reviewing will be resolved by a third independent investigator, appropriate revisions to the reviewing strategy implemented, followed by double-reviewing a further 10% until reliability on video reviewing (a kappa of 0.6 or greater) is achieved. As the other outcomes are continuous variables, the Bland-Altman method will be used to plot the difference of values reported by both reviewers against the mean value for each outcome. The limits of agreement will be assessed by the interval of ± 1.96 standard deviations (SD) of the measurement difference either side of the mean difference. The null hypothesis that there is no difference on average between both reviewers will be tested using a *t* test. The mean difference will be reported with its 95% CI. Additionally, the intraclass correlation coefficients for volumes of drugs drawn, time to drug preparation, and time to drug delivery will be assessed, assuming that raters are a sample from a larger population of possible raters. The agreement will be investigated for the data of each study period.

Finally, means and SDs will be determined for perceived stress and satisfaction scores of individuals for each questionnaire item, as well as for the UTAUT questionnaire, and reported with descriptive statistics. Pearson correlations will be computed between the HR measures obtained with the watch and the scenario phases for each of the drugs and preparation methods used. In the case of missing data, a complete case analysis will be conducted. No multiple imputations are planned. All statistical tests will be two-sided with a type-I error risk of 5%. Data analysis will be carried out using GraphPad Prism, version 7 (GraphPad Software, San Diego, CA, USA) for graph figures, Stata/IC, version 14 (StataCorp, College Station, TX, USA) for descriptive analyses, R version 2.15.2 (R Foundation, Vienna, Austria) for regression models and statistical tests, and StatXact version 11.1.0 (Cytel Studio, Cambridge, MA, USA) for exact statistical tests and exact 95% CI.

## Discussion

Despite many advances in the emergency medical field in recent years, a suboptimal quality of resuscitation is still common for both adult and pediatric patients [41]. Currently, the hospital survival rate from pediatric in-hospital cardiopulmonary arrest is 36% [41], whereas it is below 10% for pediatric out-of-hospital cardiac arrests [11, 42]. According to the American Heart Association, emergency medication such as epinephrine should be considered in children undergoing pediatric advanced life support [33, 43]. Many critical situations also require the use of drugs or substrates that require a pharmacological intervention to restore vital functions and prevent patients from deteriorating (e.g., anticonvulsive drugs, dextrose, etc.). Although much attention has been paid to in-hospital pediatric emergent medication errors which are present in almost half of all resuscitation cases [12], with significant dose deviation from the prescribed dose reported in up to 16% of the analyzed syringes [35], data regarding EMS-related prehospital medication errors and error prevention strategies are scarce [13]. However, EMS are largely exposed to opportunities for out-of-hospital medication errors, thus severely compromising patient safety. In this setting, children are likely to require immediate care [14] and resources are limited. Prehospital dosing errors affect approximately 56,000 children treated by EMS each year in the USA [6] with drugs administered outside of the proper dose range reported in up to 39.8% of more than 5500 children. Among other drugs [44], epinephrine was shown to have the highest rates of incorrect doses with 60.9% of preparations containing an error and a mean error overdose of 808%. These results are similar to others reporting that paramedics commit dosing errors 49–63% of the time, with miscalculation as a primary cause [6]. The rate of medication errors further increases in critical care environments requiring the administration of several drugs where each may have its own concentration, dose and volume [3]. Disruptive emotional anxiety and exogenous conditions encountered during prehospital pediatric resuscitation, such as challenging field environments, parental stress, and time pressure to prepare the drugs on-site are other factors that potentially add to the complexity of the process and increase the cognitive load [45, 46]. The latter has been shown to be higher and error-prone when a task is uncommon [45, 47, 48]. Paramedics have little exposure to critically ill children and lack experience to administer emergency medications at pediatric doses [49], with minimal opportunities to gain and maintain competence in this skill [13, 15]. Pediatric situations only account for about 7% of EMS calls [16, 50]. It was shown that the delivery of epinephrine to children by EMS in the USA accounted for only 3.6% of the total adult drug administration [49] and this well illustrates the fact that almost 60% of paramedics report their initial education course as deficient in pediatric-specific training [13].

Providing EMS with ready access to a weight-estimation device app and a drug-dosing guide, such as the Broselow-Luten tape (BLT), was shown to lack sufficient accuracy and information to function as a complete resuscitation aid for the prevention of a high rate of prehospital medication errors [51, 52], with epinephrine dosing errors exceeding 60–70% [44]. Even after interventions to train EMS in the use of the BLT and precalculated drug-dosing charts, medication dosing errors remain significant [49]. Underlying causes of errors include incorrect estimation of weight, incorrect use of the BLT, incorrect recall of doses, difficulty with calculations under stress, mg/kg to milligrams to milliliter conversion errors, inaccurate measurement of volumes, and failure to cross-check doses between providers [51].

Therefore, the sole expertise of paramedics helped by conventional methods is not sufficient to ensure the fast and reliable conversion and preparation of pediatric emergency IV medication. Although numerous interventions involving information technologies have been developed to improve the in-hospital security of the medication process [53, 54], there has been no trial evaluating the impact of a mobile app used by EMS to reduce prehospital medication errors, time to drug preparation and time to drug delivery during pediatric resuscitation. As most of the results obtained from simulation-based resuscitation studies agree with those obtained in real-life studies, we are confident that our results could be of great interest for a potential application in real-life situations encountered by EMS.

Our study has some limitations. First, it will be conducted during a resuscitation simulation-based scenario rather than tested in real-life situations. However, high-fidelity simulation is an essential investigative methodology to answer research questions that cannot be answered otherwise during real CPR as the diversity of patients and their diseases make such studies hard to standardize in critical situations [30]. Moreover, standardizing the scenario and the environment will help to avoid effect modifiers by limiting the influence of undesired variables on the outcomes. Second, the 5-min app training will be dispensed just before the scenario. In real life, the interval between training and actual use would probably be months. However, training with the app months before the study would unblind participants to its purpose and could create a preparation bias. Third, multiple dose calculators are available on the web or as smartphone apps, but most are not evidence-based. In our previous multicenter trial [23], the app has been validated as an efficient tool to reduce in-hospital medication errors and delays in pediatric CPR. Its selection as study comparator is, therefore, justified. Finally, the Likert-type questionnaire that will be used to measure stress has not been assessed for validity, internal consistency, reliability or generalizability. Although it cannot objectively measure the stress perceived, it can be used to measure the difference of perceived stress.

In conclusion, and to the best of our knowledge, PedAMINES is the only evidence-based mobile app to assist medical prescriptions for in-hospital pediatric resuscitation with the capacity to reduce medication errors, as well as time to drug delivery during simulated resuscitations. It remains to be determined whether the use of this app by EMS may significantly reduce medication error rates and time to drug preparation in the prehospital setting where paramedics are less exposed to pediatric resuscitation. Although the survival rate has complex and numerous components, every single minute saved in the preparation of emergency medications in the prehospital setting can lead to an increase of 9% in the survival odd [19]. As research in this area is scarce, it is anticipated that the results generated from this study will be of great importance, with the potential to change and improve pediatric prehospital emergency care practice in this vulnerable population and thus increase the survival rate.

### Trial status

The protocol version is 1.0 (29 March 2018). The trial is registered at ClinicalTrials.gov, ID: NCT03921346, 18 April 2019, https://clinicaltrials.gov/ct2/show/NCT03921346. The recruitment of subjects is expected to start mid-2019. We anticipate that the intervention will be completed in early 2020 and study results will be available in late 2020 (publication expected in early 2021).

## Supplementary information

**Additional file 1.** Standard Protocol Items: Recommendations for Interventional Trials (SPIRIT) 2013 Checklist.

**Additional file 2.** Participant informed consent form (version 1.0).

**Additional file 3.** The table describes poor or improper techniques that may lead to contamination in aseptic preparation and/or intravenous administration of emergency drugs. Adapted from Suvikas-Peltonen et al. [55].

## Data Availability

The final (anonymized) trial datasets will be accessible for investigators of the Geneva Children’s Hospital for final analyses. An anonymous copy of the final datasets underlying publications resulting from this trial will be available from the corresponding author upon reasonable and approved request.

## Abbreviations

BLT: Broselow-Luten tape
CI: Confidence interval
CONSORT: Consolidated Standards of Reporting Trials
CPR: Cardiopulmonary resuscitation
EMS: Emergency medical services
HR: Heart rate
IV: Intravenously administered
PED: Pediatric emergency department
PedAMINES: Pediatric Accurate Medication IN Emergency Situations
SD: Standard deviation
SPIRIT: Standard Protocol Items: Recommendations for Interventional Trials
UTAUT: Unified theory of acceptance and use of technology

## References

[1] Brindley PG, O’Dochartaigh D, Volney C, Ryan S, Douma MJ (2017). Time delays associated with vasoactive medication preparation and delivery in simulated patients at risk of cardiac arrest. J Crit Care.

[2] Flannery AH, Parli SE (2016). Medication errors in cardiopulmonary arrest and code-related situations. Am J Crit Care.

[3] Kaufmann J, Laschat M, Wappler F (2012). Medication errors in pediatric emergencies: a systematic analysis. Dtsch Arztebl Int.

[4] Moreira ME, Hernandez C, Stevens AD, Jones S, Sande M, Blumen JR (2015). Color-coded prefilled medication syringes decrease time to delivery and dosing error in simulated emergency department pediatric resuscitations. Ann Emerg Med.

[5] Gonzales K (2010). Medication administration errors and the pediatric population: a systematic search of the literature. J Pediatr Nurs.

[6] Hoyle JD, Davis AT, Putman KK, Trytko JA, Fales WD (2012). Medication dosing errors in pediatric patients treated by emergency medical services. Prehosp Emerg Care.

[7] Marcin JP, Dharmar M, Cho M, Seifert LL, Cook JL, Cole SL (2007). Medication errors among acutely ill and injured children treated in rural emergency departments. Ann Emerg Med.

[8] Morgan N, Luo X, Fortner C, Frush K (2006). Opportunities for performance improvement in relation to medication administration during pediatric stabilization. Qual Saf Health Care.

[9] Stucky ER (2003). American Academy of Pediatrics Committee on Drugs; American Academy of Pediatrics Committee on Hospital Care. Prevention of medication errors in the pediatric inpatient setting. Pediatrics..

[10] Kaushal R, Bates DW, Landrigan C, McKenna KJ, Clapp MD, Federico F (2001). Medication errors and adverse drug events in pediatric inpatients. JAMA..

[11] Kamarainen A (2010). Out-of-hospital cardiac arrests in children. J Emerg Trauma Shock.

[12] Porter E, Barcega B, Kim TY (2014). Analysis of medication errors in simulated pediatric resuscitation by residents. West J Emerg Med.

[13] Hoyle JD, Crowe RP, Bentley MA, Beltran G, Fales W (2017). Pediatric prehospital medication dosing errors: a national survey of paramedics. Prehosp Emerg Care..

[14] Cottrell EK, O’Brien K, Curry M, Meckler GD, Engle PP, Jui J (2014). Understanding safety in prehospital emergency medical services for children. Prehosp Emerg Care..

[15] Su E, Schmidt TA, Mann NC, Zechnich AD (2000). A randomized controlled trial to assess decay in acquired knowledge among paramedics completing a pediatric resuscitation course. Acad Emerg Med.

[16] Shah MN, Cushman JT, Davis CO, Bazarian JJ, Auinger P, Friedman B (2008). The epidemiology of emergency medical services use by children: an analysis of the National Hospital Ambulatory Medical Care Survey. Prehosp Emerg Care..

[17] Matos RI, Watson RS, Nadkarni VM, Huang HH, Berg RA, Meaney PA (2013). Duration of cardiopulmonary resuscitation and illness category impact survival and neurologic outcomes for in-hospital pediatric cardiac arrests. Circulation..

[18] Andersen LW, Berg KM, Saindon BZ, Massaro JM, Raymond TT, Berg RA (2015). Time to epinephrine and survival after pediatric in-hospital cardiac arrest. JAMA..

[19] Hansen M, Schmicker RH, Newgard CD, Grunau B, Scheuermeyer F, Cheskes S (2018). Time to epinephrine administration and survival from nonshockable out-of-hospital cardiac arrest among children and adults. Circulation..

[20] Fukuda T, Kondo Y, Hayashida K, Sekiguchi H, Kukita I (2018). Time to epinephrine and survival after paediatric out-of-hospital cardiac arrest. Eur Heart J Cardiovasc Pharmacother.

[21] Foltin GL, Richmond N, Treiber M, Skomorowsky A, Galea S, Vlahov D (2012). Pediatric prehospital evaluation of NYC cardiac arrest survival (PHENYCS). Pediatr Emerg Care.

[22] Rittenberger JC, Bost JE, Menegazzi JJ (2006). Time to give the first medication during resuscitation in out-of-hospital cardiac arrest. Resuscitation..

[23] Siebert JN, Ehrler F, Combescure C, Lovis C, Haddad K, Hugon F (2019). A mobile device application to reduce medication errors and time to drug delivery during simulated paediatric cardiopulmonary resuscitation: a multicentre, randomised, controlled, crossover trial. Lancet Child Adolesc Health.

[24] Eysenbach G, CONSORT-EHEALTH Group (2011). improving and standardizing evaluation reports of Web-based and mobile health interventions. J Med Internet Res.

[25] Cheng A, Kessler D, Mackinnon R, Chang TP, Nadkarni VM, Hunt EA (2016). International Network for Simulation-based Pediatric Innovation Research and Education (INSPIRE). Reporting guidelines for health care simulation research: extensions to the CONSORT and STROBE Statements. Simul Healthc.

[26] Chan AW, Tetzlaff JM, Gotzsche PC, Altman DG, Mann H, Berlin JA (2013). SPIRIT 2013 explanation and elaboration: guidance for protocols of clinical trials. BMJ..

[27] World Medical Association (2013). World Medical Association Declaration of Helsinki: ethical principles for medical research involving human subjects. JAMA..

[28] International Conference on Harmonisation (1999). ICH harmonised tripartite guideline. Statistical principles for clinical trials. International Conference on Harmonisation E9 Expert Working Group. Stat Med.

[29] Hagberg H, Siebert J, Gervaix A, Daehne P, Lovis C, Manzano S (2016). Improving drugs administration safety in pediatric resuscitation using mobile technology. Stud Health Technol Inform.

[30] Cheng A, Auerbach M, Hunt EA, Chang TP, Pusic M, Nadkarni V (2014). Designing and conducting simulation-based research. Pediatrics..

[31] Bottiger BW, Bernhard M, Knapp J, Nagele P (2016). Influence of EMS-physician presence on survival after out-of-hospital cardiopulmonary resuscitation: systematic review and meta-analysis. Crit Care.

[32] Siebert JN, Ehrler F, Combescure C, Lacroix L, Haddad K, Sanchez O (2017). A mobile device app to reduce time to drug delivery and medication errors during simulated pediatric cardiopulmonary resuscitation: a randomized controlled trial. J Med Internet Res.

[33] de Caen AR, Berg MD, Chameides L, Gooden CK, Hickey RW, Scott HF (2015). Part 12: pediatric advanced life support: 2015 American Heart Association guidelines update for cardiopulmonary resuscitation and emergency cardiovascular care. Circulation..

[34] Banerjee PR, Ganti L, Pepe PE, Singh A, Roka A, Vittone RA (2019). Early on-scene management of pediatric out-of-hospital cardiac arrest can result in improved likelihood for neurologically-intact survival. Resuscitation..

[35] Kozer E, Seto W, Verjee Z, Parshuram C, Khattak S, Koren G (2004). Prospective observational study on the incidence of medication errors during simulated resuscitation in a paediatric emergency department. BMJ..

[36] Maaskant JM, Vermeulen H, Apampa B, Fernando B, Ghaleb MA, Neubert A, et al. Interventions for reducing medication errors in children in hospital. Cochrane Database Syst Rev. 2015;(3):CD006208. 10.1002/14651858.CD006208.pub3 PMC10799669 25756542

[37] Rinke ML, Bundy DG, Velasquez CA, Rao S, Zerhouni Y, Lobner K (2014). Interventions to reduce pediatric medication errors: a systematic review. Pediatrics..

[38] Venkatesh V, Morris MG, Davis GB, Davis FD (2003). User acceptance of information technology: toward a unified view. MIS Q.

[39] Sealedenvelope.com. http://www.sealedenvelope.com. Accessed on 30 Oct 2018. Archived by WebCite at http://www.webcitation.org/6pj1kvMba.

[40] United States Census Bureau. URL: https://www.census.gov/geo/reference/ua/uafaq.html. Accessed: 16 Nov 2018. Archived by WebCite at http://www.webcitation.org/6y5wXS6Pv.

[41] Meaney PA, Bobrow BJ, Mancini ME, Christenson J, de Caen AR, Bhanji F (2013). Cardiopulmonary resuscitation quality: [corrected] improving cardiac resuscitation outcomes both inside and outside the hospital: a consensus statement from the American Heart Association. Circulation..

[42] Jayaram N, McNally B, Tang F, Chan PS (2015). Survival after out-of-hospital cardiac arrest in children. J Am Heart Assoc.

[43] Kleinman ME, Chameides L, Schexnayder SM, Samson RA, Hazinski MF, Atkins DL (2010). Part 14: pediatric advanced life support: 2010 American Heart Association guidelines for cardiopulmonary resuscitation and emergency cardiovascular care. Circulation..

[44] Hoyle JD, Davis AT, Putman KK, Trytko JA, Fales WD. Medication dosing errors in pediatric patients treated by emergency medical services. Prehosp Emerg Care. 2012;16(1):59-66. 10.3109/10903127.2011.614043 21999707

[45] Cushman JT, Fairbanks RJ, O’Gara KG, Crittenden CN, Pennington EC, Wilson MA (2010). Ambulance personnel perceptions of near misses and adverse events in pediatric patients. Prehosp Emerg Care..

[46] LeBlanc VR, MacDonald RD, McArthur B, King K, Lepine T (2005). Paramedic performance in calculating drug dosages following stressful scenarios in a human patient simulator. Prehosp Emerg Care..

[47] Luten R, Wears RL, Broselow J, Croskerry P, Joseph MM, Frush K (2002). Managing the unique size-related issues of pediatric resuscitation: reducing cognitive load with resuscitation aids. Acad Emerg Med.

[48] Seidel JS, Henderson DP, Ward P, Wayland BW, Ness B (1991). Pediatric prehospital care in urban and rural areas. Pediatrics..

[49] Kaji AH, Gausche-Hill M, Conrad H, Young KD, Koenig WJ, Dorsey E (2006). Emergency medical services system changes reduce pediatric epinephrine dosing errors in the prehospital setting. Pediatrics..

[50] Frey M, Trede I (2017). Swiss rescue teams in Switzerland. Swiss Health Observatory.

[51] Lammers R, Byrwa M, Fales W (2012). Root causes of errors in a simulated prehospital pediatric emergency. Acad Emerg Med.

[52] Wells M, Goldstein LN, Bentley A, Basnett S, Monteith I (2017). The accuracy of the Broselow tape as a weight estimation tool and a drug-dosing guide—A systematic review and meta-analysis. Resuscitation..

[53] Bonnabry P (2005). Information technologies for the prevention of medication errors. CHIMIA..

[54] Kahn S, Abramson EL (2019). What is new in paediatric medication safety?. Arch Dis Child.

[55] Suvikas-Peltonen E, Hakoinen S, Celikkayalar E, Laaksonen R, Airaksinen M (2017). Incorrect aseptic techniques in medicine preparation and recommendations for safer practices: a systematic review. Eur J Hosp Pharm.

