# Supplementary material for: A mobile device app to reduce prehospital medication errors and time to drug preparation and delivery by emergency medical services during simulated pediatric cardiopulmonary resuscitation: study protocol of a multicenter, prospective, randomized controlled trial
Source: Trials. 2019 Nov 20;20:634. doi: 10.1186/s13063-019-3726-4 (PMC6868759; doi:10.1186/s13063-019-3726-4)
Supplement: Supplementary file 3 — Additional file 3. The table describes poor or improper techniques that may lead to contamination in aseptic preparation and/or intravenous administration of emergency drugs. Adapted from Suvikas-Peltonen et al. [55]. [file 13063_2019_3726_MOESM3_ESM.docx]

**Additional file 3**

| Potential aseptic errors associated with the preparation of emergency drugs |
| --- |
| Lack of hand hygiene, disinfection |
| Nurses did not use gloves |
| No cleaning/disinfection of the working surface/IV tray |
| No disinfection of vial necks |
| No disinfection of phial septa or IV ports |
| Multiple use of the same needle/syringe |
| Improper needle and/or syringe handling (i.e. contact with fingers and/or surrounding environment) |
| Lack of patient skin disinfection |
| Improper IV catheter handling (i.e. contact with fingers and/or surrounding environment) |
